# Prevalence of depressive symptoms and associated factors among medical residents at four hospitals in Ecuador: a cross-sectional study

**DOI:** 10.3389/fpsyt.2026.2003488

**Published:** 2026-10-13

**Authors:** José Alejandro Valdevila Figueira, Andrés Ramírez, Indira Dayana Carvajal Parra, Samijazath Alexander Naranjo Alban, Rocío Valdevila Santiesteban, Jose A. Rodas, María José Pico Cucalón, Rodolfo Farfán

**Affiliations:** 1 Instituto de Neurociencias de Guayaquil, Guayaquil, Ecuador; 2 Universidad Ecotec, Samborondón, Ecuador; 3 Red de Investigación en Psicología y Psiquiatría (RIPYP), Guayaquil, Ecuador; 4 Carrera de Psicología Clínica, Universidad Politécnica Salesiana, Cuenca, Ecuador; 5 Universidad Católica de Santiago de Guayaquil, Guayaquil, Ecuador; 6 School of Psychology, Universidad Espíritu Santo, Samborondón, Ecuador; 7 School of Psychology, University College Dublin, Dublin, Ireland; 8 Junta de Beneficencia de Guayaquil, Guayaquil, Ecuador

**Keywords:** depressive symptoms, healthcare professionals, medical residents, mental health, physician training, PHQ-9

## Abstract

**Background:**

Medical residents face high clinical, academic, and personal demands; however, evidence regarding depressive symptoms during postgraduate medical training in Ecuador remains limited.

**Objective:**

This study aimed to estimate the prevalence of depressive symptoms and evaluate associated sociodemographic, academic, and occupational factors.

**Methods:**

A cross-sectional study using an electronic survey was conducted between May 31 and June 30, 2026, among medical residents from four Ecuadorian institutions. Of 302 eligible residents, 159 accessed the survey; 151 provided informed consent, completed the questionnaire, and were included in the analysis. Depressive symptoms were assessed using the Patient Health Questionnaire-9 (PHQ-9). Clinically relevant depressive symptoms were defined as a PHQ-9 score ≥10. Prevalence estimates were calculated with 95% confidence intervals. Associated factors were examined using univariable and multivariable logistic regression.

**Results:**

The mean age was 33.2 years (SD = 4.3); 93 participants (61.6%) were women, 82 (54.3%) were enrolled in clinical specialties, and 69 (45.7%) in surgical specialties. The prevalence of clinically relevant depressive symptoms (PHQ-9 ≥ 10) was 37.7% (95% CI: 30.4-45.7). In the adjusted model, second-year residents (aOR = 3.82; 95% CI: 1.37-10.64) and third-year residents (aOR = 4.33; 95% CI: 1.47–12.72) had higher odds of PHQ-9 scores ≥10 than first-year residents. No statistically significant associations were observed with age, sex, specialty category, monthly on-call shifts, or relationship status.

**Conclusions:**

Depressive symptoms were common among participating medical residents at four hospitals in Ecuador, with higher adjusted odds of PHQ-9 scores ≥10 among second- and third-year residents compared with first-year residents. These findings suggest the potential value of confidential mental health screening and institutional support strategies during medical residency. Longitudinal studies are needed to examine temporal relationships and identify modifiable factors within the training environment.

## Introduction

Depression is a leading cause of disability worldwide and represents a significant burden on health systems, with functional, social, and economic repercussions for affected individuals, their families, and their communities (1). Depressive disorders contribute substantially to global morbidity and are associated with impaired daily functioning, reduced productivity, and increased utilization of healthcare services (2–6).

In Latin America, the approach to addressing depression is shaped by social inequalities, sociocultural factors, and limitations regarding timely access to mental health services. Studies conducted in the region have reported a high frequency of depressive symptoms, particularly among women and urban populations (7–9). These conditions can be especially significant for groups facing intense professional and academic demands, such as healthcare professionals in training.

Medical residents represent a particularly vulnerable population due to the combination of clinical responsibilities, academic requirements, and personal demands. International evidence estimates that the prevalence of depressive symptoms in this population ranges from approximately 20% to 43%, with variations depending on the assessment tools used, program characteristics, and working conditions (10–12). Associated factors include long working hours, frequent on-call shifts, sleep deprivation, academic pressure, limited autonomy, and repeated exposure to clinically complex and emotionally taxing situations (13–15). Depressive symptoms among medical residents not only affect their individual well-being but have also been linked to reduced professional performance, a higher likelihood of medical errors, and compromised quality of care (15).

Mental health issues have also been documented among other healthcare workers, with depressive symptom prevalence rates ranging from 17.9% to 36% internationally (16, 17). In Latin America, adverse working conditions, resource shortages, and heavy clinical workloads can exacerbate this issue (18). In Ecuador, available evidence regarding medical residents remains limited and has focused primarily on specific specialties or periods associated with the COVID-19 pandemic (19, 20). Consequently, there is still a need for multicenter data examining the distribution of depressive symptoms across different specialties and years of training, as well as their relationship with sociodemographic, academic, and professional characteristics.

Assessing depressive symptoms using standardized instruments is essential for estimating their frequency and identifying groups that may require further clinical evaluation. The PHQ-9 is a brief, widely used tool with adequate psychometric properties and validated use in Latin American and Ecuadorian populations (21–24). However, it should be regarded as a screening instrument rather than a standalone diagnostic measure.

Against this backdrop, the aim of this study was to determine the prevalence and severity of depressive symptoms among medical residents across various specialties at four Ecuadorian hospitals and to analyze their association with sociodemographic, academic, and occupational factors. The findings may help identify potentially vulnerable periods and groups, as well as guide institutional strategies for detection, referral, and prevention within residency programs.

## Materials and methods

### Study design and setting

An analytical cross-sectional study was conducted using a confidential, self-administered online questionnaire. Data collection took place between May 31 and June 30, 2026. The manuscript was prepared in accordance with the recommendations of the Strengthening the Reporting of Observational Studies in Epidemiology (STROBE) statement for cross-sectional studies (25).

The study was carried out at four hospital institutions in Ecuador: a specialized neuroscience institute, a pediatric hospital, a gynecology-obstetrics hospital, and a clinical-surgical hospital. The breakdown of participants by hospital is not presented because certain combinations of center, specialty, and residency year involved a small number of participants, which could facilitate their indirect identification.

### Participants

The eligible population consisted of 302 physicians enrolled in residency or postgraduate specialization programs at the four participating institutions. A census sampling approach was used, and all 302 eligible residents at the participating institutions were invited to participate. Therefore, no *a priori* sample size calculation was performed. Participation was voluntary, and invitations were sent via institutional email.

Physicians were included if they were currently enrolled in a postgraduate training program, provided electronic informed consent, and completed the study questionnaire. Of the 302 invited residents, 159 accessed the form (52.6%). Eight participants declined to participate at the consent stage and did not complete the instruments. The final analytical sample consisted of 151 residents (50.0% of the eligible population).

### Variables and instruments

#### Depressive symptoms

Depressive symptoms over the preceding two weeks were assessed using the Spanish version of the Patient Health Questionnaire-9 (PHQ-9), previously validated in the Ecuadorian population (23). The instrument comprises nine items scored from 0 to 3, with a total score range of 0 to 27. Severity was classified as minimal/absent (0-4), mild (5-9), moderate (10-14), moderately severe (15-19), and severe (20-27).

The primary outcome was the presence of clinically relevant depressive symptoms, defined as a PHQ-9 score ≥10. A PHQ-9 score ≥5 was used in a secondary descriptive analysis to identify mild or more severe depressive symptoms. These categories were used for screening purposes rather than for clinical diagnosis. Internal consistency of the PHQ-9 was estimated using Cronbach’s alpha.

#### Sociodemographic, academic, and professional variables

Sociodemographic variables (age, women/men, marital status, and number of children) and academic/professional variables (medical specialty, specialty category (clinical or surgical), residency year [R1-R4], number of monthly on-call shifts, and time since medical graduation) were collected. For regression analyses, marital status was recoded into “with a partner” (married or in a domestic partnership) and “without a partner” (single or divorced). Individual specialties were described descriptively and grouped into clinical and surgical categories for the multivariable models due to the limited size of some categories. Time since graduation was categorized as ≤3 years, 4–6 years, and ≥7 years.

### Procedure

Data were collected via an electronic form administered through Google Forms over a period of approximately one month. Invitations were sent to medical residents via institutional email and reinforced by academic authorities and residency program directors. Because the electronic survey was configured to require responses for all mandatory fields prior to submission, there were no missing data for any of the variables included in the final analytical dataset.

Before accessing the questionnaire, participants received information regarding the study objectives, the voluntary nature of participation, confidentiality, and data handling; they then provided electronic informed consent. A unique access code was implemented to minimize duplicate responses; this code was stored separately and was not included in the analysis dataset.

Participation was voluntary and entailed no academic, professional, or clinical consequences. Electronic reminders were sent during the data collection period. Prior to statistical analysis, the dataset was reviewed to identify duplicates, inconsistent values, invalid ranges, and missing data.

Prior to the start of data collection, the protocol was evaluated and approved by the Human Research Ethics Committee of Luis Vernaza Hospital (Resolution 40-EO-CEISH-HLV-2026). The study was conducted in accordance with the principles of autonomy, beneficence, non-maleficence, justice, privacy, and confidentiality, as well as the 2024 revision of the Declaration of Helsinki (26).

Informed consent was obtained electronically prior to the administration of the instruments. No names, identification numbers, addresses, or other direct identifiers were collected. Data were stored in password-protected files, and access was restricted to the research team.

To guarantee strict anonymity and encourage honest reporting of sensitive psychiatric symptoms, specific institutional identifiers for the four participating hospitals were not recorded in the analytical dataset.

### Statistical analysis

Continuous variables were summarized using means and standard deviations, and categorical variables using frequencies and percentages. Prevalence estimates were calculated with 95% confidence intervals using the Wilson score method. Differences in the proportion of participants with a PHQ-9 score ≥ 10 across categorical characteristics were evaluated using Pearson’s chi-square tests or Fisher’s exact tests, as appropriate.

To explore factors associated with clinically relevant depressive symptoms (PHQ-9 ≥ 10), univariable and multivariable logistic regression analyses were conducted. Covariates in the primary multivariable model—age, sex, specialty category, residency year, monthly on-call shifts, and partner status—were specified *a priori* based on theoretical relevance and established literature in graduate medical education, irrespective of univariable significance. Given the sample size (N = 151) and the number of outcome events (n = 57), this multivariable model was treated as exploratory, estimating eight predictor degrees of freedom (~7.1 events per parameter). Additional candidate variables (number of children, time since graduation) were restricted to univariable screening to avoid further parameter burden. Furthermore, because age, residency year, and time since graduation exhibit substantial conceptual and empirical overlap, time since graduation was excluded from the multivariable model to prevent multicollinearity, allowing the model to focus on residency year as the primary exposure of interest. For continuous predictors (age and monthly on-call shifts), the assumption of linearity on the logit scale was evaluated and confirmed using the Box-Tidwell procedure. To assess model stability against potential overfitting and parameter saturation, a parsimonious sensitivity analysis was conducted specifying residency year as the sole predictor.

Because the primary outcome was common (> 10%), odds ratios can overestimate relative associations. Therefore, a complementary modified Poisson regression with robust variance estimators (sandwich standard errors) was conducted to estimate adjusted prevalence ratios (aPRs). Model fit for the primary logistic regression was evaluated using the likelihood-ratio test and McFadden’s and Nagelkerke’s pseudo-R^2^ statistics. Because the objective of the multivariable model was strictly to explore associated factors rather than to develop a predictive tool, these pseudo-R^2^ metrics were included solely to quantify the modest proportion of variance explained by the measured covariates and to underscore the likely contribution of unmeasured institutional and personal factors.

pAll statistical tests were two-sided, and a p-value <.05 was considered statistically significant. Analyses were performed in R (version 4.6.1; R Core Team, 2026), utilising the packages tidyverse (version 2.0.0), broom (version 1.0.12), DescTools (version 0.99.60), car (version 3.1.5), psych (version 2.6.3), sandwich (version 3.1.1), and lmtest (version 0.9.40).

## Results

### Sociodemographic characteristics of participants

Of the 302 invited resident physicians, 159 accessed the electronic form. Of these, 151 provided informed consent, completed the questionnaire, and were included in the analyses, representing 50.0% of the eligible population. Eight individuals declined to participate and did not access the study instruments.

The mean age of the participants was 33.2 years (SD = 4.3; range: 25–58), and 93 (61.6%) were women. The majority were single (n = 86; 57.0%) and had no children (n = 91; 60.3%) (**Table 1**).

**Table 1:** Sociodemographic, academic, and occupational characteristics.

Characteristic	Value
Age (years), *M* (*SD*)	33.17 (4.34)
Sex, *n* (%)	
Men	58 (38.4)
Women	93 (61.6)
Marital status, *n* (%)	
Single	86 (57.0)
Married	44 (29.1)
Divorced	13 (8.6)
Cohabiting	8 (5.3)
Number of children, *n* (%)	
0	91 (60.3)
1	34 (22.5)
2	20 (13.2)
3	6 (4.0)
Specialty category, *n* (%)	
Clinical	82 (54.3)
Surgical	69 (45.7)
Residency year, *n* (%)	
R1	44 (29.1)
R2	43 (28.5)
R3	33 (21.9)
R4	31 (20.5)
Monthly on-call shifts, *M* (*SD*)	7.61 (2.63)
Time since graduation, *n* (%)	
≤ 3 years	22 (14.6)
4–6 years	63 (41.7)
≥ 7 years	66 (43.7)
PHQ-9 total score, *M* (*SD*)	7.92 (5.78)

N = 151. M, mean; SD, standard deviation; PHQ-9, Patient Health Questionnaire-9; R1–R4, first through fourth year of residency training. Percentages may not sum to exactly 100% due to rounding.

### Academic and professional characteristics

Eighty-two participants (54.3%) were enrolled in clinical specialties, and 69 (45.7%) in surgical specialties. The sample included 44 first-year residents (R1; 29.1%), 43 second-year residents (R2; 28.5%), 33 third-year residents (R3; 21.9%), and 31 fourth-year residents (R4; 20.5%). The mean number of monthly on-call shifts was 7.61 (SD = 2.63), with a median of 8.00 (IQR: 8.00–8.00; range: 0–16). In total, 129 participants (85.4%) had graduated from medical school four or more years prior. Anaesthesiology was the most frequent individual specialty (n = 35; 23.2%), followed by paediatrics and internal medicine, with 17 participants each (11.3%) (**Supplementary Table S1**).

### Prevalence and severity of depressive symptoms

The mean PHQ-9 score was 7.92 (SD = 5.78), with a median of 7.00 (IQR: 4.00–12.00; range: 0–24). Internal consistency in this sample was adequate (Cronbach’s α = .906). The primary outcome of clinically relevant depressive symptoms (PHQ-9 ≥ 10) was identified in 57 participants, corresponding to a prevalence of 37.7% [95% CI: (30.4, 45.7)]. In secondary descriptive analyses, 102 participants exhibited scores of at least mild severity (PHQ-9 ≥ 5), yielding a prevalence of 67.5% (95% CI: [59.7, 74.5]). The distribution across all five screening severity categories is displayed in **Figure 1**).

**Figure 1:**
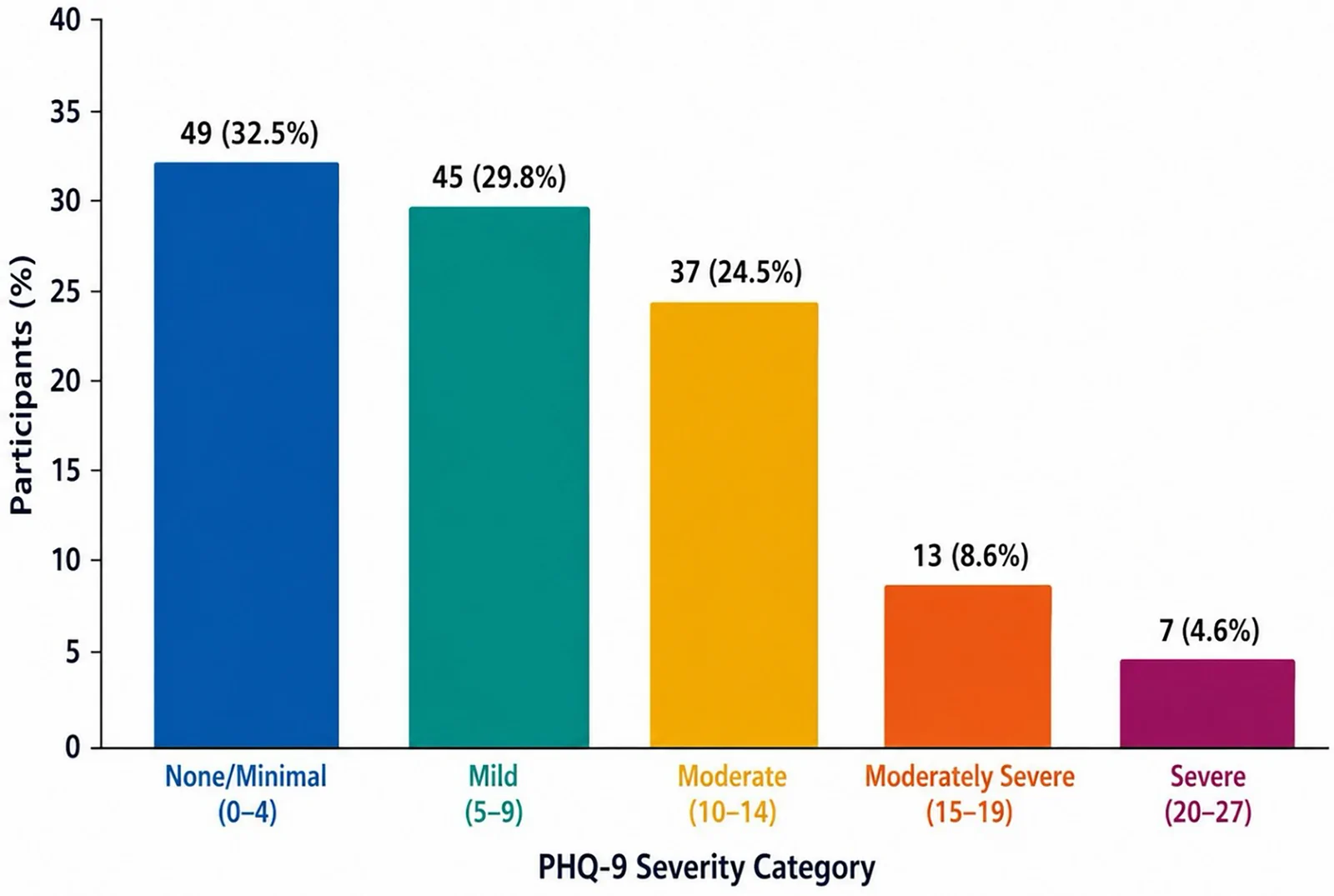
Distribution of depressive symptom severity according to the PHQ-9 among resident physicians (n = 151). Labels above the bars indicate the absolute frequency and percentage of participants in each PHQ-9 severity category. Categories were defined as none/minimal (0-4), mild (5-9), moderate (10-14), moderately severe (15-19), and severe (20-27). These categories represent screening-based symptom severity levels and do not constitute clinical diagnoses.

### Prevalence according to participant characteristics

The prevalence of PHQ-9 scores ≥10 was 31.0% among men and 41.9% among women, with no statistically significant difference (p = .179). Similarly, prevalence was 42.7% among residents in clinical specialties and 31.9% among those in surgical specialties (p = .173). Prevalence differed significantly across residency years (p = .034): 22.7% for R1, 46.5% for R2, 51.5% for R3, and 32.3% for R4. A statistically significant difference was also observed across marital status categories (p = .046), although this comparison should be interpreted with caution due to sparse cells. No statistically significant differences were observed based on the presence of children (p = .904) or time elapsed since medical graduation (p = .639) (**Table 2**).

**Table 2:** Prevalence of clinically relevant depressive symptoms (PHQ-9 ≥ 10) across participant characteristics.

Characteristic	Total, n	Cases, n	Prevalence (%)	Statistical test	p
Sex				Pearson’s χ^2^	.179
Men	58	18	31.0		
Women	93	39	41.9		
Specialty category				Pearson’s χ^2^	.173
Clinical	82	35	42.7		
Surgical	69	22	31.9		
Residency yearh				Pearson’s χ^2^	.034
R1	44	10	22.7		
R2	43	20	46.5		
R3	33	17	51.5		
R4	31	10	32.3		
Marital status				Fisher’s exact	.046
Single	86	31	36.0		
Married	44	18	40.9		
Divorced	13	2	15.4		
Cohabiting	8	6	75.0		
Children				Pearson’s χ^2^	.904
None	91	34	37.4		
At least one	60	23	38.3		
Time since graduation				Pearson’s χ^2^	.639
≤3 years	22	9	40.9		
4–6 years	63	21	33.3		
≥7 years	66	27	40.9		

N = 151. Cases represent participants with a Patient Health Questionnaire-9 (PHQ-9) score ≥ 10. Prevalence corresponds to the proportion of cases within each category. Two-sided p-values were obtained using Pearson’s chi-square test without continuity correction or Fisher’s exact test (with Monte Carlo simulation for marital status due to expected cell counts < 5). R1–R4 = first through fourth year of residency training.

### Factors associated with clinically relevant depressive symptoms

In univariable analyses, second-year (R2) and third-year (R3) residents demonstrated higher odds of presenting with PHQ-9 scores ≥ 10 compared with first-year (R1) residents [OR = 2.96, 95% CI: (1.19, 7.69), p = .022; and OR = 3.61, 95% CI: (1.38, 9.94), p = .010, respectively]. The association for fourth-year (R4) residents was not statistically significant compared to R1 (OR = 1.62, 95% CI: [0.57, 4.60], p = .360).

In the primary multivariable model, which adjusted for age, sex, specialty category, monthly on-call shifts, and partner status, the association between training year and depressive symptoms persisted. Both R2 and R3 residents maintained higher adjusted odds of clinically relevant symptoms compared with R1 residents [aOR = 3.82, 95% CI: (1.40, 11.00), p = .010; and aOR = 4.33, 95% CI: (1.51, 13.20), p = .008, respectively]. An omnibus Wald test confirmed the global significance of residency year an associated factor within the adjusted model (χ^2^(3) = 9.45, p = .024).

To provide effect estimates that are more easily interpreted given the commonality of the outcome (37.7% prevalence), a modified Poisson regression with robust standard errors was performed. This complementary analysis confirmed the findings of the logistic regression: R2 and R3 residents retained a significantly higher prevalence of clinically relevant depressive symptoms compared with R1 residents [aPR = 2.29, 95% CI: (1.19, 4.38), p = .013; and aPR = 2.39, 95% CI: (1.23, 4.64), p = .010, respectively].

No statistically significant independent associations were observed for age, sex, specialty category, monthly on-call shifts, or partner status. Although residents in surgical specialties exhibited lower adjusted odds of clinically relevant depressive symptoms compared with those in clinical specialties, this difference did not reach statistical significance and was characterised by wide confidence intervals [aOR = 0.49, 95% CI: (0.22, 1.05), p = .070], reflecting substantial statistical uncertainty rather than evidence of equivalence. Time since graduation and the number of children were evaluated in exploratory univariable analyses to prevent parameter saturation and demonstrated no statistically significant associations with the outcome (**Table 3**).

**Table 3:** Logistic regression analyses of factors associated with clinically relevant depressive symptoms (PHQ-9 ≥ 10).

Factor	OR [95% CI]	p	aOR [95% CI]	p
Primary model variables				
Age, per 5-year increase	1.23 [0.84, 1.82]	.297	1.02 [0.65, 1.62]	.919
Women vs. men	1.60 [0.81, 3.25]	.180	1.76 [0.84, 3.78]	.140
Surgical vs. clinical specialty	0.63 [0.32, 1.22]	.174	0.49 [0.22, 1.05]	.070
Residency year				
R2 vs. R1	2.96 [1.19, 7.69]	.022	3.82 [1.40, 11.00]	.010
R3 vs. R1	3.61 [1.38, 9.94]	.010	4.33 [1.51, 13.20]	.008
R4 vs. R1	1.62 [0.57, 4.60]	.360	1.92 [0.59, 6.28]	.275
On-call shifts/month, per shift	0.99 [0.87, 1.12]	.811	1.05 [0.92, 1.21]	.480
With vs. without a partner	1.71 [0.86, 3.42]	.124	1.82 [0.85, 3.94]	.124
Exploratory univariable variables				
Number of children, per child	1.09 [0.74, 1.59]	.658	—	—
*Time since graduation*				
4–6 years vs. ≤3 years	0.72 [0.27, 2.00]	.523	—	—
≥7 years vs. ≤3 years	1.00 [0.38, 2.73]	>.999	—	—

OR, odds ratio (univariable); aOR, adjusted odds ratio (multivariable); CI, confidence interval; R1–R4, first through fourth year of residency training. The primary multivariable model was specified *a priori* and included age, sex, specialty category, residency year, monthly on-call shifts, and partner status. Children and time since graduation were evaluated via exploratory univariable regression only to avoid parameter saturation and multicollinearity. Reference categories: men, clinical specialty, R1, without a partner, and ≤3 years since graduation. For the omnibus Wald test evaluating residency year in the multivariable model, p = .024.

### Multivariable model performance and sensitivity analysis

The primary multivariable model exhibited modest explanatory performance, accounting for a limited proportion of variance (McFadden’s R^2^ = .087; Nagelkerke’s R^2^ = .148).

Given the event-to-parameter ratio in the primary model, a parsimonious sensitivity analysis was conducted restricting the predictor set exclusively to residency year. In this restricted model, the magnitude and direction of the associations remained highly stable: R2 residents [OR = 2.96, 95% CI: (1.19, 7.69), p = .022] and R3 residents [OR = 3.61, 95% CI: (1.38, 9.94), p = .010] continued to demonstrate significantly elevated odds relative to R1 residents. The direction and statistical significance of the associations were preserved in the parsimonious analysis.

## Discussion

This study identified a high frequency of depressive symptoms among medical residents at four Ecuadorian hospitals. The primary epidemiological finding was a 37.7% prevalence of clinically relevant depressive symptoms, defined as a PHQ-9 score ≥10. The main finding was the association between the year of training and the outcome: second-and third-year residents showed higher adjusted odds of PHQ-9 scores ≥10 compared to first-year residents, after controlling for age, sex, specialty category, number of monthly on-call shifts, and relationship status. No statistically significant independent associations were observed for age, sex, specialty category, monthly on-call shifts, or partner status.

The mean age of the sample was similar to that reported among Peruvian residents, although the present study had a higher representation of women (27). The inclusion of residents from clinical and surgical specialties across all four years of training allowed for the exploration of differences related to academic progression. However, the lack of an association with time since graduation suggests that prior professional experience does not necessarily offer protection against the demands of residency.

### Prevalence and severity of depressive symptoms

The 37.7% prevalence of PHQ-9 scores ≥10 was higher than the 28.8% estimated by Mata et al. in their systematic review and meta-analysis of over 17, 000 medical residents (10). However, that review showed considerable heterogeneity, with estimates ranging from 20.9% to 43.2%, depending on the instrument, the cutoff point, and the characteristics of the programs evaluated. Consequently, the observed prevalence falls within the international range, albeit at the upper end.

Direct comparisons with other studies must consider the specific instruments and diagnostic thresholds used, as varying screening cut-offs and diagnostic interviews are not directly interchangeable. The results are consistent with studies conducted in Ecuador. Sánchez et al. reported a depression prevalence of 38.8% among Ecuadorian medical residents, a figure virtually equivalent to that found in the present study. Similarly, Mackenzie-Ugarte et al. reported mild to moderate depressive symptoms in 61.9% of paediatric and paediatric surgery residents. Although definitions and categories are not entirely comparable, these findings support the existence of a high burden of depressive symptoms during postgraduate medical training in the country.

In Mexico, Fernández-Ortega et al. identified moderately severe or severe symptoms in 14.3% of family medicine residents, a proportion close to the 13.2% observed in our sample when combining both categories. More variable results have been reported in other contexts. Alshardi and Farahat reported depressive symptoms in 75.8% of residents in Saudi Arabia, whereas Alva-Diaz et al. found a prevalence of 14.6% among Peruvian residents. These comparisons highlight the necessity of standardising assessment tools, as studies using the PHQ-2, PHQ-9, or differing thresholds yield divergent epidemiological data.

The findings also align with longitudinal studies documenting an increase in depressive symptoms following the start of medical training. Sen et al. observed that the proportion of interns meeting criteria for depression rose from 3.9% before the internship to 25.7% during that period (12). Similarly, Mata et al. estimated a mean absolute increase of 15.8% after the start of residency (10). Although the cross-sectional design of the present study does not allow for the establishment of temporality or causality, this evidence suggests that training conditions may contribute to psychological distress.

The mean PHQ-9 score corresponded to a mild symptom level; however, 20 participants exhibited moderately severe or severe symptoms. This proportion highlights the need for programs to have accessible mechanisms for clinical assessment and care in place. The PHQ-9 is a screening tool and does not establish a diagnosis of depressive disorder on its own; therefore, high scores should be complemented by a clinical interview assessing symptom duration, functional impairment, differential diagnoses, and suicide risk.

The PHQ-9 demonstrated good internal consistency, although self-reporting can be subject to recall bias, social desirability bias, and under-reporting linked to professional stigma. This is a relevant issue, as physicians in training tend to underutilize mental health services. Guille et al. found that while 42.5% of interns exhibited depressive symptoms, only 22.7% sought treatment (28). The primary barriers included a lack of time, access difficulties, a preference for coping with problems independently, and concerns regarding confidentiality. Therefore, screening will be insufficient unless accompanied by services that are accessible, confidential, and independent of academic and professional evaluations.

### Year of training and other associated factors

Training stage was the sole predictor demonstrating a statistically significant association with clinically relevant depressive symptoms, confirmed by an omnibus Wald test for the categorical variable (χ^2^(3) = 9.45, p = .024). Crucially, the observed relationship did not follow a linear trajectory across training progression. Instead, it exhibited a non-monotonic, inverted-U distribution: compared with first-year residents, adjusted odds peaked in the second [aOR = 3.82, 95% CI: (1.40, 11.00), p = .010] and third years [aOR = 4.33, 95% CI: (1.51, 13.20), p = .008], before declining to non-significant differences by the fourth year [aOR = 1.92, 95% CI: (0.59, 6.28), p = .275].

Because R2 and R3 represent distinct trainee cohorts rather than longitudinal observations of the same individuals across time, we hypothesize that this intermediate elevation reflects a period of concentrated psychological vulnerability during mid-residency. Second- and third-year trainees encounter substantially expanded clinical workloads, acute patient-care responsibilities, and supervisory roles over junior trainees, yet often possess limited administrative autonomy, inflexible scheduling, and incomplete clinical mastery. By contrast, the symptom attenuation observed among fourth-year residents could reflect consolidated clinical competence, greater autonomy, or anticipation of graduation. Alternatively, this decline may be influenced by survivor bias, wherein trainees with unmanageable distress interrupt or delay their training before reaching senior status. Because educational climate, feedback quality, and attrition were not directly measured, and given the cross-sectional design, these interpretations remain exploratory hypotheses requiring longitudinal verification.

The observed pattern differs partially from the meta-analysis by Mata et al., which did not identify consistent differences based on the level of training (10). This discrepancy could reflect variations across programs, specialties, healthcare systems, and institutional cultures. The relationship between the academic year and depressive symptoms likely depends on the interaction between the demands of each level, available supervision, and program organization.

Although women showed a higher prevalence and adjusted OR, the association did not reach statistical significance. The direction of the effect aligns with studies identifying greater vulnerability among women in medical training. Sánchez et al. found an association between female sex and comorbid depression and anxiety among physicians in training in Ecuador (29), while Sen et al. identified female sex as a predictor of a greater increase in symptoms during internship (12). The lack of statistical significance in our sample could be attributed to limited statistical precision and the failure to measure factors such as domestic responsibilities, discrimination, harassment, and social support. Among Mexican residents, Enriquez Estrada et al. documented higher levels of workplace victimization among women and an association between these experiences, anxiety, and impaired mental quality of life (30).

Although residents in surgical specialties showed lower adjusted odds of depressive symptoms compared with those in clinical specialties, this difference was not statistically significant and presented wide confidence intervals (aOR = 0.49, 95% CI: [0.22, 1.05], p = .070). This interval width indicates considerable estimation uncertainty rather than evidence of equivalence between specialty tracks. The broad, dichotomous grouping into clinical and surgical domains was necessary to avoid sparse cells, but it may have masked nuanced differences across subspecialties with disparate workloads, operating theatre demands, and supervisory environments. Our findings are consistent with the heterogeneous results reported across the literature, including the meta-analysis by Mata et al. (10), which did not identify systematic differences across broad specialty types.

### Workload and personal characteristics

The monthly number of on-call shifts was not associated with PHQ-9 scores ≥10. This result contrasts with studies linking higher workloads to depressive symptoms. Alva-Diaz et al. found that each additional hour worked per day was associated with an 11% increase in the prevalence of depressive symptoms (27), while Pereira-Lima et al. observed that long work hours were linked to a greater increase in symptoms (31).

One possible explanation is that the number of on-call shifts is an incomplete measure of work exposure. Residents with the same monthly number of shifts may differ regarding shift duration, patient volume and complexity, sleep interruptions, and recovery time. Furthermore, the sample’s high concentration around exactly eight monthly shifts (IQR: 8–8), reflecting highly standardised institutional residency schedules, severely limited the variance necessary to detect associations when modelling the variable linearly.

The relationship between workload and mental health may also depend on the organizational characteristics of the programs. Pereira-Lima et al. found that long work hours and lower-quality educational feedback were associated with a greater increase in symptoms, whereas a favorable learning experience was linked to fewer symptoms (31). Among hospital workers in Guayaquil, Alvarado et al. observed that excessive work effort was linked to higher emotional exhaustion, while perceived reward could have a protective effect (32). Although burnout and depression are distinct constructs, they may share associated factors such as overload, low autonomy, inadequate supervision, and a lack of recognition.

Age and relationship status did not show independent associations in the adjusted model. The number of children and the time elapsed since graduation were also not associated with the outcome in the univariate analyses. These results do not rule out their potential relevance, as their effects could depend on the quality of social support, shared domestic responsibilities, economic stability, and institutional flexibility-variables that were not assessed.

The multivariable model was statistically significant overall but exhibited modest explanatory performance (McFadden’s R^2^ = 0.087, Nagelkerke’s R^2^ = 0.148). This modest performance indicates that substantial variability in depressive symptoms remains unexplained by the measured sociodemographic and training-related covariates, warranting caution when interpreting the identified associations. Unmeasured factors—such as personal or family history of depression, objective working hours, sleep quality, adverse clinical events, workplace mistreatment, social support, and program satisfaction—likely account for a considerable portion of this residual variance. Sen et al. identified a history of depression, neuroticism, early adverse experiences, working hours, and adverse medical events as factors associated with increased symptoms (12). In Ecuador, Sánchez et al. found that health-promoting behaviors were associated with lower odds of depression and anxiety (29).

### Clinical and institutional implications

The results support the implementation of institutional strategies to detect and address mental health issues within residency programs. Although second- and third-year residents may constitute priority groups, monitoring should extend to all levels of training. Periodic screening using validated instruments must be complemented by clinical assessment, protocols for managing suicide risk, and confidential services that are separate from academic and professional evaluation processes.

Individual interventions -such as self-care, emotional regulation, or mindfulness programs- can be useful but should not replace organizational measures. Busireddy et al. found that reducing specific work demands can decrease emotional exhaustion, although the effects on other dimensions of well-being vary (33). Consequently, programs should combine individual strategies with structural changes addressing work schedules, recovery periods, the distribution of responsibilities, the quality of supervision, educational feedback, and mechanisms for preventing and reporting harassment.

### Strengths and limitations

The study’s strengths include the inclusion of residents from diverse specialties and all four years of training, the use of a widely established screening instrument, the presentation of severity categories and confidence intervals, and the analysis of both unadjusted and adjusted associations.

Several limitations should be considered. First, the cross-sectional design precludes establishing causality or temporality. Second, the PHQ-9 is a screening instrument rather than a diagnostic interview, and self-reporting may be subject to recall and social desirability biases. Third, statistical power for multivariable modelling was constrained by the overall sample size (N = 151) and event count (n = 57). With eight predictor degrees of freedom, the event-to-variable ratio was approximately 7.1, resulting in relatively wide confidence intervals and modest explanatory power (McFadden’s R^2^ = .087). Although parsimonious sensitivity analyses confirmed that the associations for intermediate residency years remained stable when covariates were removed, our multivariable findings remain exploratory and require replication in larger cohorts. Fourth, the number of monthly on-call shifts did not capture their duration, weekly working hours, sleep quality, or rotation intensity. Fifth, potentially relevant factors - such as burnout, anxiety, sleep quality, workplace mistreatment, financial pressure, social support, substance use, mental health history, and institutional characteristics - were not assessed. Evidence from network analyses suggests that depression, anxiety, fatigue, sleep disturbances, self-efficacy, and burnout are interrelated among medical residents (34).

A major limitation of this study is the 50.0% participation rate (151 of 302 eligible residents), which introduces potential selection bias. Participation in a mental health survey may itself be intrinsically linked to psychological status, workload, available time, or professional stigma. This non-participation may have influenced the estimates in two potential directions: the prevalence could be underestimated if residents experiencing severe depressive symptoms or extreme workload lacked the time or energy to complete the survey; conversely, it could be overestimated if those experiencing psychological distress were more motivated to participate due to the survey’s personal relevance. Furthermore, if non-participation varied systematically by residency year or specialty, the magnitude of the observed associations may have been attenuated or inflated. Consequently, the reported 37.7% prevalence and the corresponding associations must be interpreted cautiously and cannot be definitively generalised to all medical residents in Ecuador. Seventh, although this was a multicentre study, institutional identifiers were intentionally not collected to protect participant anonymity. Consequently, we could not statistically adjust for hospital-level clustering or inter-centre differences in workload, supervision, or scheduling practices, leaving the potential for residual confounding.

Finally, the adjusted model was exploratory and based on 57 outcome events. The limited number of events and the width of some confidence intervals reduce the precision of the estimates; therefore, the results require replication in larger samples and across a greater number of institutions.

## Conclusions

Depressive symptoms were common among the resident physicians evaluated; however, given the 50.0% response rate and the potential for selection bias, this 37.7% prevalence cannot necessarily be considered representative of all medical residents in Ecuador. Within this sample, the highest adjusted odds of clinically relevant depressive symptoms (PHQ-9 ≥ 10) were observed during the second and third years of training. Although the cross-sectional design precludes establishing causality, these findings suggest that the intermediate residency years may represent a potentially vulnerable period, a hypothesis that requires confirmation in longitudinal studies, alongside the implementation of integrated preventive and support strategies. Longitudinal studies involving a larger number of hospitals and more comprehensive measurements of personal, work-related, and organizational factors are needed to examine temporal trajectories, assess institutional differences, and identify modifiable associated factors.

## Data Availability

The raw data supporting the conclusions of this article will be made available by the authors, without undue reservation.

## References

[1] World Health Organization . Depressive Disorder (Depression). Geneva: World Health Organization (2025). Available online at: https://www.who.int/news-room/fact-sheets/detail/depression (Accessed September 10, 2026).

[2] Santomauro DF Herrera AMM Shadid J Zheng P Ashbaugh C Pigott DM . Global prevalence and burden of depressive and anxiety disorders in 204 countries and territories in 2020 due to the COVID-19 pandemic. Lancet. (2021) 398:1700–12. doi: 10.1016/s0140-6736(21)02143-7 34634250 PMC8500697

[3] Ferrari AJ Charlson FJ Norman RE Patten SB Freedman G Murray CJ . Burden of depressive disorders by country, sex, age, and year: results from the Global Burden of Disease study 2010. PloS Med. (2013) 10:e1001547. Available online at: https://journals.plos.org/plosmedicine/article?id=10.1371/journal.pmed.1001547. 10.1371/journal.pmed.1001547 PMC3818162 24223526

[4] Vigo D Thornicroft G Atun R . Estimating the true global burden of mental illness. Lancet Psychiatry. (2016) 3:171–8. Available online at: https://www.thelancet.com/journals/lanpsy/article/PIIS2215-0366(15)00505-2/abstract?showall=true%3D. 10.1016/S2215-0366(15)00505-2 26851330

[5] Kessler RC de Jonge P Shahly V van Loo HM Wang PSE Wilcox MA . Epidemiology of depression. In: Handbook of Depression. The Guilford Press, New York (2014). p. 7–24.

[6] Krebber AMH Buffart LM Kleijn G Riepma IC De Bree R Leemans CR . Prevalence of depression in cancer patients: a meta-analysis of diagnostic interviews and self-report instruments. Psicooncología. (2014) 23:121–30. Available online at: https://onlinelibrary.wiley.com/doi/full/10.1002/pon.3409. 10.1002/pon.3409 PMC4282549 24105788

[7] Pan American Health Organization . Epidemiology of Mental Disorders in Latin America and the Caribbean. Pan American Health Organization (2009). Available online at: https://iris.paho.org/handle/10665.2/740 (Accessed September 10, 2026).

[8] Kohn R Levav I De Almeida JM Vicente B Andrade L Caraveo-Anduaga JJ . Mental disorders in Latin America and the Caribbean: a public health priority. Pan Am J Public Health. (2005) 18:229–40. Available online at: https://europepmc.org/article/med/16354419. 10.1590/s1020-49892005000900002 16354419

[9] Alarcón RD . Mental health and mental health care in Latin America. World Psychiatry. (2003) 2:54–6. Available online at: https://pmc.ncbi.nlm.nih.gov/articles/PMC1525063/. PMC1525063 16946892

[10] Mata DA Ramos MA Bansal N Khan R Guille C Di Angelantonio E . Prevalence of depression and depressive symptoms among resident physicians: A systematic review and meta-analysis. JAMA. (2015) 314:2373–83. Available online at: https://jamanetwork.com/journals/jama/fullarticle/2474424. 10.1001/jama.2015.15845 PMC4866499 26647259

[11] Rotenstein LS Ramos MA Torre M Segal JB Peluso MJ Guille C . Prevalence of depression, depressive symptoms, and suicidal ideation among medical students: A systematic review and meta-analysis. JAMA. (2016) 316:2214–36. Available online at: https://jamanetwork.com/journals/jama/fullarticle/2589340. 10.1001/jama.2016.17324 PMC5613659 27923088

[12] Sen S Kranzler HR Krystal JH Speller H Chan G Gelernter J . A prospective cohort study investigating factors associated with depression during medical internship. Arch Gen Psychiatry. (2010) 67:557–65. doi: 10.1001/archgenpsychiatry.2010.41 20368500 PMC4036806

[13] Dyrbye LN West CP Satele D Boone S Tan L Sloan J . Burnout among U.S. medical students, residents, and early career physicians relative to the general U.S. population. Acad Med. (2014) 89:443–51. doi: 10.1097/acm.0000000000000134 24448053

[14] Shanafelt TD Hasan O Dyrbye LN Sinsky C Satele D Sloan J . Changes in burnout and satisfaction with work-life balance in physicians and the general U.S. working population between 2011 and 2014. Mayo Clinic Proc. (2015) 90:1600–13. doi: 10.1016/j.mayocp.2015.08.023 26653297

[15] Fahrenkopf AM Sectish TC Barger LK Sharek PJ Lewin D Chiang VW . Rates of medication errors among depressed and burnout residents: Prospective cohort study. BMJ. (2008) 336:488–91. doi: 10.1136/bmj.39469.763218.be 18258931 PMC2258399

[16] Olaya B Pérez-Moreno M Bueno-Notivol J Gracia-García P Lasheras I Santabárbara J . Prevalence of depression among healthcare workers during the COVID-19 outbreak: A systematic review and meta-analysis. J Clin Med. (2021) 10:3406. doi: 10.3390/jcm10153406 34362188 PMC8348388

[17] Fernández R Sikhosana N Green H Halcomb EJ Middleton R Alananzeh I . Anxiety and depression among healthcare workers during the COVID-19 pandemic: A systematic umbrella review of the global evidence. BMJ Open. (2021) 11:e054528 34548373. 10.1136/bmjopen-2021-054528 PMC8458002

[18] Muñoz-Ortega S Santamaría-Guayaquil D Pluas-Borja J Alvarado-Villa G Sandoval V Alvarado R . Mental health in healthcare workers post-COVID-19: A Latin American review and insights into personalized management strategies. J Personalized Med. (2024) 14:680. doi: 10.3390/jpm14070680 39063934 PMC11278025

[19] Mackenzie-Ugarte D Arboleda-Barrera C Carrillo L Espinoza de los Monteros A . Prevalence of depressive symptoms among medical residents at a pediatric hospital in Ecuador: a cross-sectional study. Gaceta Médica la Junta Beneficencia Guayaquil. (2025) 3:1–10. doi: 10.61708/8hx21t35

[20] Cuartero-Castañer ME Von Wyl A Hastings J Schönfeld P Meléndez JC Moncayo C . Professional quality of life, engagement, and self-care in healthcare professionals in Ecuador during the COVID-19 pandemic. Healthcare. (2021) 9:515. Available online at: https://www.mdpi.com/2227-9032/9/5/515. 10.3390/healthcare9050515 PMC8146458 33946629

[21] Kroenke K Spitzer RL Williams JBW . The PHQ-9: Validity of a brief depression severity measure. J Gen Internal Med. (2001) 16:606–13 11556941 doi: 10.1046/j.1525-1497.2001.016009606.x PMC1495268

[22] Levis B Benedetti A Thombs BD . Accuracy of Patient Health Questionnaire-9 (PHQ-9) for screening to detect major depression: Individual participant data meta-analysis. BMJ. (2019) 365:l1476. Available online at: https://www.bmj.com/content/365/bmj.l1476. 10.1136/bmj.l1476 PMC6454318 30967483

[23] Quiñonez-Freire C Vara MD Tomás JM Baños RM . Psychometric properties of the Spanish version of the Patient Health Questionnaire-9 in users of the Ecuadorian public health care system. Rev Latinoamericana Psicología. (2021) 53:210–7. Available online at: http://www.scielo.org.co/scielo.php?pid=S0120-05342021000100210&script=sci_arttext&tlng=en.

[24] López-Guerra VM López-Núñez C Vaca-Gallegos SL Torres-Carrión PV . Psychometric properties and factor structure of the Patient Health Questionnaire-9 as a screening tool for depression among Ecuadorian college students. Front Psychol. (2022) 13:813894. Available online at: https://www.frontiersin.org/journals/psychology/articles/10.3389/fpsyg.2022.813894/full. 10.3389/fpsyg.2022.813894 PMC9105228 35572338

[25] von Elm E Altman DG Egger M Pocock SJ Gøtzsche PC Vandenbroucke JP . The Strengthening the Reporting of Observational Studies in Epidemiology (STROBE) statement: guidelines for reporting observational studies. Lancet. (2007) 370:1453–7. doi: 10.1016/s0140-6736(07)61602-x 18064739

[26] World Medical Association . WMA Declaration of Helsinki: Ethical Principles for Medical Research Involving Human Participants. Adopted by the 75 Th WMA General Assembly, Helsinki, Finland, October 2024. Ferney-Voltaire (France): World Medical Asossiation (2024). Available online at: https://www.wma.net/policies-post/wma-declaration-of-helsinki/ (Accessed September 10, 2026).

[27] Alva-Diaz C Nieto-Gutiérrez W Taype-Rondan A Timaná-Ruiz R Herrera-Añazco P Jumpa-Armas D . Association between daily working hours and the presence of depressive symptoms among medical residents in Peru. Rev Colomb Psiquiatr. (2021) 50:22–8. doi: 10.1016/j.rcp.2019.03.007 33648691

[28] Guille C Speller H Laff R Epperson CN Sen S . Utilization and barriers to mental health services among depressed medical interns: A prospective multisite study. J Grad Med Educ. (2010) 2:210–4. doi: 10.4300/JGME-D-09-00086.1 21975622 PMC2941380

[29] Sánchez X Cruz A Cortez MD Jimbo-Sotomayor R Escalante S Mera D . Health-promoting behaviors are associated with lower depression and anxiety in Ecuadorian medical residents: a cross-sectional study. Front Public Health. (2026) 13:1715795. doi: 10.3389/fpubh.2025.1715795 41602085 PMC12832361

[30] Enriquez Estrada VM Antonio-Villa NE Bello-Chavolla OY Cuevas-García CF Vargas Gutiérrez PL Noriega ISYC . Assessment of psychological terror and its impact on mental health and quality of life in medical residents at a reference medical center in Mexico: A cross-sectional study. PloS One. (2023) 18:e0295138. doi: 10.1371/journal.pone.0295138 38055666 PMC10699592

[31] Pereira-Lima K Gupta RR Guille C Sen S . Residency program factors associated with depressive symptoms in internal medicine interns: A prospective cohort study. Acad Med. (2019) 94:869–75. doi: 10.1097/ACM.0000000000002567 30570500 PMC6538448

[32] Alvarado LE Bretones FD Rodríguez JA . The effort-reward model and its effect on burnout among nurses in ecuador. Front Psychol. (2021) 12:760570. doi: 10.3389/fpsyg.2021.760570 34887811 PMC8648575

[33] Busireddy KR Miller JA Ellison K Ren V Qayyum R Panda M . Efficacy of interventions to reduce resident physician burnout: A systematic review. J Grad Med Educ. (2017) 9:294–301. doi: 10.4300/JGME-D-16-00372.1 28638506 PMC5476377

[34] Li J Li T Xiao C Duan Y Jiäng Y Geng W . Association between burnout and physiological-psychological-social health domains among Chinese resident physicians: a network analysis approach. BMC Health Serv Res. (2025) 25:1156. doi: 10.1186/s12913-025-13200-y 40885990 PMC12398008

