# Supplementary material for: Prevalence of depressive symptoms and associated factors among medical residents at four hospitals in Ecuador: a cross-sectional study
Source: Front Psychiatry. 2026 Oct 13;17:2003488. doi: 10.3389/fpsyt.2026.2003488 (PMC13710397; doi:10.3389/fpsyt.2026.2003488)
Supplement: Supplementary file 1 [file Table1.pdf]

Supplementary Materials

Supplementary Table S1

Distribution of participants by medical specialty (n = 151)

Medical specialty	n	%
Anaesthesiology	35	23.2
Paediatrics	17	11.3
Internal medicine	17	11.3
Obstetrics and gynaecology	14	9.3
General surgery	11	7.3
Family medicine	10	6.6
Psychiatry	9	6.0
Radiology and imaging	9	6.0
Critical care medicine	7	4.6
Paediatric surgery	7	4.6
Dermatology	3	2.0
Neonatology	3	2.0
Neurology	2	1.3
Sports medicine	2	1.3
Ophthalmology	2	1.3
Gastroenterology	1	0.7
Emergency and disaster medicine	1	0.7
Cardiology	1	0.7

Note. n = 151. Values are presented as absolute frequencies and percentages of the total sample. Each participant was classified according to their current medical specialty. Percentages may not total exactly 100% because of rounding.
